# The mediating role of problem solving in the relationship between emotional intelligence and mental well-being

**DOI:** 10.3389/fpsyg.2025.1687583

**Published:** 2026-01-05

**Authors:** Ramazan Erdoğan, Büşra Özcan, Oktay Kızar, Mustafa Kızılkoca, İrem Kaptangil Çalışır, Eyüp Bozkurt, Fatih Mehmet Uğurlu, Suriye Tan, Meryem Koçal

**Affiliations:** 1 Faculty of Sport Sciences, Munzur University, Tunceli, Türkiye; 2 School of Physical Education and Sports, Siirt University, Siirt, Türkiye; 3 Vocational School of Social Sciences, Bitlis Eren University, Bitlis, Türkiye; 4 Faculty of Education, Firat University, Elazığ, Türkiye; 5 Faculty of Sport Sciences, Firat University, Elazığ, Türkiye; 6 Kuşadası Sports Football Club, Aydın, Türkiye

**Keywords:** emotional intelligence, mental well-being, problem solving, university students, mediator effect

## Abstract

**Background:**

This study aims to examine the relationship between emotional intelligence and mental well-being among university students and to reveal the mediating role of problem-solving skills in this relationship.

**Methods:**

Of these students, 707 participated, and the Emotional Intelligence Trait Scale, the Warwick-Edinburgh Psychological Well-being Scale, and the Problem-Solving Inventory were used as data collection tools. The SPSS software was used for data analysis.

**Results:**

Emotional intelligence appears to have a significant effect on mental well-being, and emotional intelligence directly significantly predicts problem-solving skills, but mental well-being does not have a significant effect on problem-solving skills. Emotional intelligence appears to have a direct effect on problem-solving, and mental well-being mediates this effect. The explanatory power of the model is moderate in terms of the role of emotional intelligence in predicting mental well-being and more limited in terms of its role in predicting problem-solving skills.

**Conclusion:**

In conclusion, emotional intelligence strengthens individuals’ mental well-being and directly positively impacts their problem-solving skills. However, mental well-being does not play a mediating role in the problem-solving process. This suggests that the direct effect of emotional intelligence is primary in improving problem-solving skills, and mental well-being does not serve as a decisive mediating factor in this process. Therefore, interventions aimed at strengthening emotional intelligence can be effective both in enhancing individuals’ mental well-being and in directly supporting problem-solving skills.

## 1 Introduction

The rapidly evolving social, economic, and technological landscape of the 21st century has fundamentally transformed the experiences and expectations of young adults, particularly university students. In this dynamic context, evaluating students solely on their academic performance is insufficient to capture the complexity of their developmental needs. Beyond cognitive achievements, students’ psychological resilience, emotional regulation, adaptive coping strategies, and overall life satisfaction have emerged as critical indicators of their holistic adjustment and success in academic, social, and personal domains (Fiorilli et al., 2020; Regehr et al., 2013). The contemporary university environment, characterized by heightened competition, academic demands, and rapid social changes, necessitates that students cultivate a robust set of psychological resources to navigate these multifaceted challenges effectively.

Among these psychological resources, emotional intelligence (EI) has garnered significant attention as a pivotal factor contributing to students’ psychological and social functioning. Defined broadly as the ability to perceive, understand, regulate, and utilize emotions in oneself and others (Salovey and Mayer, 1990; Petrides et al., 2016), emotional intelligence encompasses competencies such as self-awareness, emotional regulation, empathy, and interpersonal skills. Theoretical frameworks, such as Mayer and Salovey’s four-branch model of EI and Petrides’ trait emotional intelligence approach, provide a comprehensive understanding of how these abilities facilitate adaptive behaviors and decision-making in complex social and academic environments (Batool and Siddiqui, 2020; Drigas and Papoutsi, 2018). Empirical studies consistently demonstrate that individuals with higher emotional intelligence exhibit superior self-regulatory capacities, employ more adaptive coping strategies in response to stress, and report higher levels of subjective well-being, life satisfaction, and overall mental health (Zeidner et al., 2012; Sánchez-Álvarez et al., 2016; Martins et al., 2010).

Closely related to emotional intelligence is the concept of mental well-being, a multidimensional construct reflecting not only the presence of positive emotions but also the capacity to derive meaning, maintain purposeful engagement, and establish harmonious relationships within one’s social environment (Ryff and Keyes, 1995; Keyes, 2002). Mental well-being is particularly salient for university students, as this developmental period often involves navigating identity formation, academic pressures, and life transitions, which collectively impact psychological resilience and adaptive functioning (Zhang and Bartol, 2010; Arslan et al., 2021). Higher levels of emotional intelligence are positively associated with enhanced mental well-being, suggesting that emotionally intelligent individuals are better equipped to interpret and respond constructively to stressors, maintain social relationships, and engage in behaviors that promote psychological flourishing (Schutte et al., 2007; Cazan and Năstasă, 2015; Singh and Sharma, 2021).

Beyond these direct effects, recent literature emphasizes the role of cognitive and behavioral competencies in shaping the pathways from emotional intelligence to mental well-being. Problem-solving skills represent a critical dimension of these competencies, reflecting the capacity to identify, analyze, and implement effective strategies in response to complex or stressful situations (Heppner and Petersen, 1982; Girgin, 2018). Problem-solving is not only a learnable skill but also a modifiable trait, making it a prime target for psychoeducational interventions aimed at enhancing student resilience. Evidence indicates that emotional intelligence can influence the development and application of problem-solving skills, which in turn positively affect decision-making, stress management, and adaptive functioning (Sinclair, 2019; Cassady et al., 2025). For instance, emotionally intelligent individuals are more likely to approach problems with a reflective and analytical mindset, consider alternative strategies, and evaluate potential consequences before acting, thereby reducing maladaptive responses and promoting psychological well-being (El Khatib and Alawadhi, 2024).

Furthermore, problem-solving skills have been shown to serve as a mediating mechanism linking emotional intelligence to mental well-being. Students who effectively utilize problem-solving strategies are better able to cope with academic pressures, social challenges, and life transitions, which contributes to higher life satisfaction, reduced psychological distress, and enhanced adaptive functioning (Heppner, 2008; Cassady et al., 2025). This mediating role aligns with theoretical models of resilience and self-regulation, which posit that cognitive-behavioral skills can amplify the positive effects of emotional competencies on mental health outcomes.

The practical implications of understanding this mediating pathway are substantial. Universities increasingly recognize the need for evidence-based interventions that promote not only academic achievement but also psychological resilience, emotional regulation, and adaptive coping. Programs designed to strengthen emotional intelligence and problem-solving skills can contribute to students’ overall well-being, enhance their capacity to manage stress, and improve academic performance (Zhang et al., 2025; Eisenbeck et al., 2022). This is particularly relevant in the post-COVID-19 era, where students face heightened psychological pressures, disrupted routines, and increased uncertainty, all of which underscore the necessity of cultivating holistic psychological resources.

Given this theoretical and empirical background, the present study aims to investigate the relationship between emotional intelligence and mental well-being among university students, while examining the mediating role of problem-solving skills. By clarifying the interplay between these constructs, this research seeks to provide insights into the mechanisms through which emotional competencies translate into enhanced well-being. Furthermore, the findings are expected to inform the development of targeted interventions and student support programs that foster adaptive coping strategies, promote resilience, and enhance overall life satisfaction. Ultimately, such evidence-based approaches can contribute to the cultivation of well-rounded, psychologically resilient students capable of navigating the complexities of modern academic and social environments.

## 2 Materials and methods

### 2.1 Research model

This study investigates the impact of athletes’ emotional intelligence levels on their problem-solving skills. The mediating role of mental well-being in this effect was also examined. The study employed the correlational screening model, a quantitative research method used to identify relationships between two or more variables and their associated increases and decreases (Christensen et al., 2015). The theoretical hypotheses and model to be tested in the study are presented below.

*H1:* Emotional intelligence positively affects individuals’ problem-solving skills.

*H2:* Emotional intelligence positively affects individuals’ mental well-being levels.

*H3:* Mental well-being positively affects individuals’ problem-solving skills.

*H4:* Mental well-being mediates the effect of emotional intelligence on problem-solving skills.

**Figure 1:**
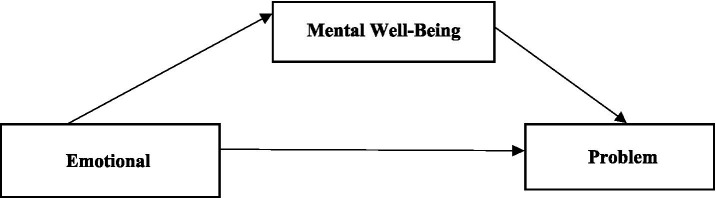
Research model.

Data were collected between February and April 2025. The study was conducted in accordance with the principles of the Declaration of Helsinki. Necessary permissions were obtained from the corresponding authors who adapted the scales used in the study. The study was approved by the Siirt University Ethics Committee at its meeting dated February 3, 2025, numbered 1191. Before participating in the study, the volunteers were informed about the study. Written and verbal consent was then obtained from the volunteers (see Figure 1).

### 2.2 Research group

In this study, multiple regression analysis was conducted using G*Power software in order to determine the statistical power of the sample size. According to the analysis results, it was determined that a minimum of 215 participants were needed to obtain significant results with 95% power (1 − *β* = 0.95), 5% significance level (*α* = 0.05) and medium effect size (f^2^ = 0.10). However, considering the possibility of data loss or missing data suggested by Suresh and Chandrashekara (2012), it was deemed appropriate to increase the sample size by approximately 10% and accordingly, the study group consisted of a total of 707 participants. This sample size is sufficient to obtain statistically significant results. It also exceeds the minimum number of observations recommended by Hayes (2022) for mediation analyses. The participants of the study consisted of 494 male (age = 20.15 ± 1.06) and 213 female (age = 19.67 ± 0.89) students studying in the faculties of sports sciences at Siirt University and Fırat University. The inclusion criteria for the study were; Participants were defined as being an undergraduate student, being physically and mentally healthy, understanding the purpose of the study, providing informed consent, and accepting participation voluntarily. However, students with a history of chronic disease, serious disability within the last 6 months, receiving psychiatric treatment, or any health problem that could affect physical performance were excluded from the study. Participants’ demographic information was recorded for basic variables such as age, gender, and year of education, and data accuracy was checked before analyses. All processes were conducted in a way that prioritized the rights and safety of research participants, confidentiality of data was ensured, and participants were informed that they could withdraw from the study at any time. The study was conducted in accordance with the fundamental principles of the Declaration of Helsinki, which sets ethical standards in human research, and written informed consent was obtained from the participants.

Missing data analysis was performed for each variable, and some missing values were identified in the PSI (Psychological Scale Name) variable. Missing data were handled using appropriate strategies based on the distribution of the relevant variables and the extent of missingness. Since the amount of missing data in this study was below 5%, analyses were conducted using listwise deletion (excluding the analysis with the full data set), preserving model integrity. Missing data analysis was performed for each variable. Since the amount of missing data was below 5%, the listwise deletion method was used, as recommended by Little and Rubin (2019). Because the rate of missing data was low (≤5%), these data did not present a statistically significant bias. Alternatively, a control analysis using the expectation–maximization method found that the results did not change significantly. The regression analysis for the PSI variable found that the explained variance (R^2^) was only 0.076. This low explanatory power was considered a limitation of the study. Therefore, it is recommended that future studies increase explanatory power by including additional variables in the model. This result indicates that the total effect of the independent variables on the PSI was limited. Therefore, it should be emphasized that the model’s explanatory power for the PSI is low, and the practical significance of the findings should not be overstated. Furthermore, the low R^2^ value suggests that variables that could have an impact on the PSI but were not included in the model (e.g., cognitive ability, academic stress, social support) were overlooked. Including such variables in the model in future research will contribute to a more comprehensive explanation of the PSI.

Ethics committee approval was obtained prior to data collection, and all participants volunteered to participate in the study. Participants were assured that their data would be used solely for scientific purposes.

### 2.3 Research ethics

The necessary permissions for this research were passed by the ethics committee with the decision of the Siirt University Scientific Research and Publication Ethics Committee dated February 3, 2025, numbered 1191.

### 2.4 Data collection tools

#### 2.4.1 Problem solving inventory (PSI)

Developed by Heppner and Petersen in 1982 and adapted into Turkish by Taylan in 1990, the scale is a self-report measure that measures an individual’s perceptions and approaches to problem solving (Taylan, 1990; Heppner and Petersen, 1982). It consists of 35 items on a six-point Likert-type scale ranging from 1 (strongly disagree) to 6 (strongly agree). However, items 9, 22, and 29 are excluded from the scoring, and items 1, 2, 3, 4, 11, 13, 14, 15, 17, 21, 25, 26, 30, and 34 are reverse scored. These items are assumed to represent adequate problem-solving skills. The scale consists of three subscales: Self-Confidence in Problem Solving (SPS): Contains 11 items (5, 10, 11, 12, 19, 23, 24, 27, 33, 34, and 35). This dimension reflects the individual’s belief in his or her ability to solve new problems. The score range is between 11 and 66. Approach-Avoidance (APA): Contains 16 items (1, 2, 4, 6, 7, 8, 13, 15, 16, 17, 18, 20, 21, 28, 30, and 31). This subscale reflects the individual’s tendency to actively strive or avoid the problem-solving process. The score range is 16–96. Personal Control (PC): This dimension consists of six items (13, 14, 25, 26, 27, and 32). This dimension measures an individual’s capacity to control themselves in problematic situations. The score range is between 5 and 30 (Bandura, 1999). The total score obtained from the scale ranges from 32 to 192. Low total scores indicate that the individual’s problem-solving skills are effective, while high scores indicate that the individual perceives himself or herself as inadequate in problem-solving.

#### 2.4.2 Warwick-Edinburgh mental well-being scale (MWS)

Developed by Tennant and colleagues (2007), it was adapted into Turkish by Keldal (2015). The scale consists of 14 items and is a 5-point Likert-type scale, ranging from 1 (Never) to 5 (Always). Possible scores range from 14 to 70. Higher scores indicate a higher level of mental well-being. The internal consistency of the Turkish version of the scale is quite high; the Cronbach’s Alpha reliability coefficient was reported as 0.89 (Keldal, 2015). This value demonstrates that the scale reliably measures mental well-being.

#### 2.4.3 Emotional intelligence trait scale–short form (EIT-SF)

The Emotional Intelligence Trait Scale–Short Form (EIT-SF), developed by Petrides and Furnham (2001), was adapted into Turkish by Deniz et al. (2013). The EIT-SF is a scale developed to determine an individual’s self-perception of their emotional competence. The 30-item scale is a 7-point Likert-type measurement tool designed to measure overall emotional intelligence. The scale consists of subscales designated as “Subjective Well-Being,” “Self-Control,” “Emotionality,” and “Sociability.” In reliability analyses, the Cronbach’s alpha coefficients for the EIT-SF were found to be 0.72 for the Well-Being factor, 0.70 for Self-Control, 0.66 for Emotionality, 0.70 for Sociability, and 0.81 for the entire scale. The test–retest reliability coefficient was found to be 0.86.

Validity and reliability analyses of the scales used in our study were performed; The internal consistency of the scales used in the study was assessed using Cronbach’s alpha coefficients. *α* = 0.88 for the EIT scale, α = 0.82 for the MWS, and α = 0.90 for the PSI. All values above 0.70 indicate a high level of reliability for the scales. Validity and reliability analyses of all scales were retested in these ranges and are presented together with the original studies.

#### 2.4.4 Data analysis

Before proceeding with data analysis, outlier and missing data analyses were conducted using SPSS 27.0 software. Data normality was assessed based on Mahalanobis distances, Z values, and skewness and kurtosis coefficients. As stated by George and Mallery (2019), skewness and kurtosis values between −2 and +2 indicate that the data meet the assumption of normal distribution. Furthermore, linear relationships between variables were examined through scatter plots, and no deviations were observed in the distributions. Tolerance and VIF (Variance Inflation Factor) values were analyzed to assess multicollinearity. The results did not indicate multicollinearity because tolerance values were >0.82 and VIF values were >1.22 for all independent variables. The research hypotheses were tested using PROCESS Macro, developed by Hayes (2022). In this context, Model 4 was used to evaluate the mediation effect to better understand the indirect relationships among the study variables. The bootstrap resampling method was applied in the regression analyses, and a sampling size of 5,000 was chosen (Hayes et al., 2017). The significance of the mediation effect was assessed by ensuring that the values within the 95% confidence interval (CI) did not include zero. Preacher and Hayes (2008) and Hayes et al. (2017) state that this is sufficient to demonstrate that the mediation effect is statistically significant.

## 3 Results

### 3.1 *T* test for differences between groups, correlation and descriptive statistics

According to Table 1, male participants’ mean scores for problem solving, emotional intelligence, and mental well-being were significantly higher than female participants (*p* < 0.05). This result suggests that male students have higher perceptions or skill levels in these variables. However, the direction of the differences may depend on cultural or sample structure; therefore, caution should be exercised when making generalizations.

**Table 1:** *T*-test analyses by gender.

Scales	Gender	*n*	Mean	SD	*t*	*p*
Problem solving	Male	494	121.03	8.27	2.970	0.003*
	Female	213	119.10	6.99		
Emotional intelligence	Male	494	59.47	11.69	3.294	0.001*
	Female	213	56.09	14.22		
Mental well-being	Male	494	42.19	8.58	3.611	0.000*
	Female	213	39.53	9.87		

*p < 0.05, **p < 0.01.

According to Table 2, no statistically significant differences were found between licensed and non-licensed athletes in terms of problem solving, emotional intelligence, and mental well-being (*p* > 0.05). This result suggests that athletic status does not have a decisive effect on these variables.

**Table 2:** *T*-test analyses based on licensed athlete status.

Scales	Licensed athlete status	*n*	Mean	SD	*t*	*p*
Problem solving	Yes	294	120.71	8.29	0.743	0.458
	No	413	120.26	7.70		
Emotional intelligence	Yes	294	57.94	11.20	−0.905	0.366
	No	413	58.81	13.50		
Mental well-being	Yes	294	41.31	8.07	−0.183	0.855
	No	413	41.44	9.72		

*p < 0.05, **p < 0.01.

An examination of Table 3 reveals statistically significant relationships between the variables. The analysis revealed moderate positive relationships between problem solving and emotional intelligence (*r* = 0.269, *p* < 0.001), between mental well-being (*r* = 0.218, *p* < 0.001), and between emotional intelligence and mental well-being (*r* = 0.628, *p* < 0.001).

**Table 3:** Correlations between variables and descriptive analyses.

Variables	1	2	3	X	SD	Skewness	Kurtosis
1. Problem solving	1			46.29	10.68	0.400	0.595
2. Emotional ıntelligence	0.269**	1		23.90	7.21	0.490	0.146
3. Mental well-being	0.218**	0.629**	1	32.45	6.53	0.439	0.466

*p < 0.05, **p < 0.01.

Table 4 results indicate significant and positive relationships between the variables (*p* < 0.01). A moderate relationship (*r* = 0.269) was found between emotional intelligence and problem solving, and a strong relationship (*r* = 0.629) was found between emotional intelligence and mental well-being. Furthermore, the low-to-moderate positive relationship (*r* = 0.218) between mental well-being and problem solving suggests that individuals in psychological well-being are more effective at problem solving. These findings suggest that emotional intelligence is an important psychological resource that supports both mental well-being and problem solving skills.

**Table 4:** Full correlation matrix with *N* per cell.

Variable	1	2	3
1. Problem solving	1	0.269** (*N* = 707)	0.218** (*N* = 707)
2. Emotional intelligence	0.269** (*N* = 707)	1	0.629** (*N* = 707)
3. Mental well-being	0.218** (*N* = 707)	0.629** (*N* = 707)	1

*N* per cell = 707 (all available data used). *p* < 0.01 for **.

According to Table 5, male students’ mean scores for problem solving (M = 46.5), emotional intelligence (M = 24.0), and mental well-being (M = 32.8) are slightly higher than those for female students (M = 45.8, 23.7, and 31.9, respectively). While these differences are not large, they suggest that male students generally have higher levels of perception or skill in these variables. However, the limited differences suggest that gender may have a weaker determining effect on these variables.

**Table 5:** Means and standard deviations by gender (*N* = 707; Male = 494, Female = 213).

Variable	Male (*n* = 494)	Female (*n* = 213)
Problem solving	M = 46.5, SD = 10.6	M = 45.8, SD = 10.7
Emotional intelligence	M = 24.0, SD = 7.2	M = 23.7, SD = 7.2
Mental well-being	M = 32.8, SD = 6.5	M = 31.9, SD = 6.5

M = Mean, SD = Standard deviation values are illustrative; replace with your exact means/SDs.

Table 6 results indicate significant and positive relationships among the variables (*p* < 0.05). A moderate positive relationship (*r* = 0.45) was found between emotional intelligence (EIT) and mental well-being (MWS). Furthermore, a low positive relationship (*r* = 0.17) was observed between emotional intelligence and problem solving (PSI) and a very low but significant relationship (*r* = 0.08) was observed between mental well-being and problem solving. These results suggest that emotional intelligence is linked to both well-being and problem-solving skills, but that the impact of mental well-being on problem solving is limited.

**Table 6:** Histograms / Q-Q plots.

	EIT	MWS	PSI
EIT	707	–	–
MWS	0.45** (*N* = 707)	707	–
PSI	0.17** (*N* = 707)	0.08* (*N* = 707)	707

**p < 0.001: stronger significance. *p < 0.05: significant.

According to Table 7, emotional intelligence (EIT) has significant and positive effects on both mental well-being (MWS) and problem solving (PSI). The effect of emotional intelligence on mental well-being is quite strong (B = 0.452, *β* = 0.63, *p* < 0.001), indicating that as emotional intelligence levels increase, individuals’ psychological well-being also increases significantly. The effect of mental well-being on problem solving is weaker but significant (B = 0.070, β = 0.08, *p* = 0.048). In addition, the direct effect of emotional intelligence on problem solving (B = 0.138, β = 0.15, *p* < 0.001) was found to be significant. Considering the total effect (B = 0.169), emotional intelligence affects problem solving skills both directly and partially indirectly, but the mediating effect is limited.

**Table 7:** Mediation analysis results (*N* = 707).

Paths	Unstandardized B	S. E.	95% CI (LLCI – ULCI)	Standardized β	*p*
a: EIT → MWS	0.452	0.021	0.411–0.493	0.63	<0.001
b: MWS → PSI	0.070	0.040	0.009–0.150	0.08	0.048
c’: EIT → PSI (direct effect)	0.138	0.024	0.080–0.196	0.15	<0.001
c: EIT → PSI (total effect)	0.169	0.024	0.121–0.217	0.18	<0.001

According to Table 8, the intercept values of the model represent the baseline levels of the variables. The constant coefficient for mental well-being (MWS) is B = 14.940, which represents the average well-being of individuals in the model, holding other variables constant. The constant coefficient for the problem-solving (PSI) variable is B = 109.464, with a 95% confidence interval between 105.523 and 112.404. These results demonstrate that the model performs significantly and consistently overall, and the margins of error are within acceptable limits.

**Table 8:** Intercepts.

Variable	B	S.E.	95% CI
M (MWS)	14.940	1.260	11.136–15.581
Y (PSI)	109.464	1.497	105.523–112.404

According to Table 9, the model’s explanatory power (R^2^) is 39.5% for mental well-being (MWS) and 7.6% for problem solving (PSI). This finding indicates that emotional intelligence explains a significant portion of the variance in mental well-being, but its explanatory power for problem-solving skills is more limited. The *F* values for both models are statistically significant (*p* < 0.001), demonstrating that the regression models are generally meaningful and valid.

**Table 9:** Model fit statistics.

Outcome variable	*R* ^2^	*F*	*p*
M (MWS)	0.395	460.687	<0.001
Y (PSI)	0.076	29.115	<0.001

a = Effect of EIT on MWS (mediation path); b = Effect of MWS on PSI; c’ = Direct effect of EIT on PSI; c = Total effect of EIT on PSI (c = c’ + a*b); 95% CI = Bootstrapped 95% confidence interval (5,000 samples).

**Figure 2:**
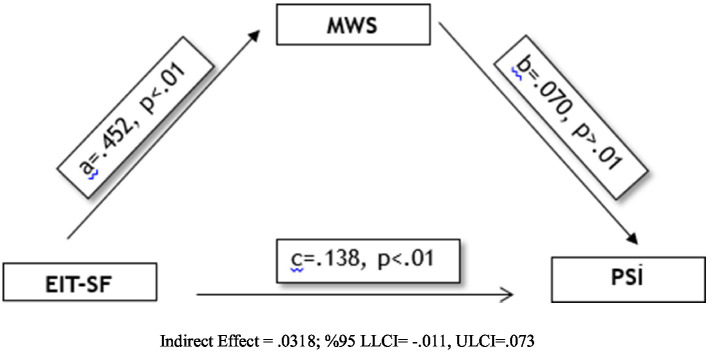
The effect of emotional intelligence on mental well-being in the regression model.

According to the results of regression analysis based on the bootstrap method, the effect of emotional intelligence on mental well-being was found to be significant (*b* = 0.452, SE = 0.021, *p* < 0.001, LLCI = 0.411, ULCI = 0.493) (see Table 10; Figure 2). The explanatory power of these variables in the model was 39.5% (R^2^ = 0.395). On the other hand, the direct effect of emotional intelligence on problem-solving skills was also significant (*b* = 0.138, SE = 0.024, *p* < 0.001, LLCI = 0.080, ULCI = 0.196). However, the effect of mental well-being on problem solving was not found to be significant (*b* = 0.070, SE = 0.040, *p* = 0.086, LLCI = −0.009, ULCI = 0.150). The overall explanatory power of this model was 7.6% (R^2^ = 0.076). Since the confidence interval for the indirect effect (*a* × *b* = 0.0318) obtained with the bootstrap method includes zero (BootLLCI = −0.011, BootULCI = 0.073), the indirect effect of emotional intelligence on problem solving is not statistically significant. These findings suggest that emotional intelligence directly predicts problem solving skills, but mental well-being does not mediate this relationship (Table 5).

**Table 10:** Results of regression analysis regarding mediation test (*n* = 707).

Forecast variables	Outcome variables							
	M (MSI-intermediary exchange)				Y (PSI-dependent variable)			
		*b*	*S.E.*	LLCI–ULCI		*b*	*S.E.*	LLCI–ULCI
X (EIT)	*a*	0.452**	0.021	0.411/0.493	*c^’^*	0.138**	0.024	0.080/0.196
M (MWS)	*–*	–	–	–	*b*	0.070	0.040	0.009/0.150
Fixed	*I_M_*	14.9400	1.26	11.136/15.581	*I_Y_*	109.464	1.497	105.523/112.404
	*R*^2^ = 0.395				*R^2^* = 0.076			
	*F* = 460.687; *p* < 0.001				*F* = 29.115; *p* < 0.001			

*p < 0.05, **p < 0.01; S.E., Standard Error; b = Unstandardized beta coefficients.

## 4 Discussion and conclusions

The findings of the study revealed that university students’ emotional intelligence levels positively impact both their mental well-being and problem-solving skills. The table revealed significant differences by gender across all three variables. Male students reported higher levels of problem-solving skills [t(705) = 2.97, *p* = 0.003], emotional intelligence [t(705) = 3.29, *p* = 0.001], and mental well-being [t(705) = 3.61, *p* < 0.001] than female students. The higher mean scores for male students compared to females across all three variables may be related to the sample consisting of sports science students. Sports education generally supports cognitive-emotional skills such as coping with stress, self-control, and decision-making. In Yiğiter’s (2018) study, the relationship between social problem-solving skills and sports participation was examined in terms of gender; no significant difference was found in problem-solving skills between individuals who participated in sports and those who did not. However, when comparing male and female students, it was observed that participation in sports had a more significant impact on problem-solving skills in males, supporting the contribution of sports participation to psychosocial development. Carmeli et al. (2009) stated that individuals with high emotional intelligence are more likely to experience higher levels of psychological well-being and that this relationship may also differ by gender. The study examined the relationships between emotional intelligence and the sub-dimensions of psychological well-being (self-acceptance, life satisfaction, somatic complaints, and self-esteem). It was found that women’s higher levels of emotional intelligence compared to men had a stronger impact, particularly on self-acceptance and life satisfaction. Yilmaz and Yiğit (2020) examined the problem-solving skills of students at the faculty of sports sciences in terms of various personal variables and found that these skills did not differ significantly based on gender and sports participation. However, the overall findings revealed that sports participation had positive effects on students’ problem-solving skills, indicating that participation in sports supports cognitive and psychosocial development in both female and male students (Wismath and Zhong, 2014). This suggests that while female students may initially have lower self-confidence in problem-solving, they can often bridge this gap with appropriate educational support and experience. Therefore, it can be argued that emotional intelligence and self-efficacy-based training can be particularly effective in strengthening female students’ problem-solving skills. This result is consistent with numerous studies in the literature. For example, Safara et al. (2023) and Sánchez-Álvarez et al. (2020) emphasized that emotional intelligence positively impacts psychological well-being through self-awareness, seeking social support, and stress management. The study found that emotional intelligence has a high impact on mental well-being (*b* = 0.452, *p* < 0.001), demonstrating the role of emotional regulation skills in enhancing students’ subjective well-being.

Similarly, the direct effect of emotional intelligence on problem-solving skills was found to be significant (*b* = 0.138, *p* < 0.001). This finding is consistent with research conducted by Cassady et al. (2022) on university students, which showed that emotional intelligence is associated with individuals’ mental well-being and promotes solution-focused approaches. This suggests that more emotionally stable students are able to develop more effective coping strategies in stressful academic or social situations.

On the other hand, the lack of statistical significance in the mediating effect of mental well-being on problem-solving (*b* = 0.070, *p* = 0.086) is a striking finding of the study. This result indicates that although well-being provides a general state of psychological well-being at the individual level, it may not be a direct trigger for problem-solving behaviors (Zhou and Yao, 2020). Similarly, Liu et al. (2024) and Arslan and Allen (2022) suggested that well-being alone may not be a determinant of problem-solving skills and should be evaluated together with cognitive and behavioral strategies.

Based on these findings, it can be concluded that emotional intelligence should be considered a protective factor, particularly in the academic and social lives of university students. Furthermore, it can be predicted that educational interventions aimed at strengthening emotional intelligence skills will also support students’ ability to cope with stress, make decisions, and generate solutions. In this context, it is recommended that university guidance services focus on the development of emotional intelligence in their psychoeducational programs.

The research results revealed that university students’ emotional intelligence levels have significant and positive effects on both mental well-being and problem-solving skills. These findings align with studies by researchers such as Sánchez-Álvarez et al. (2020) and Safara et al. (2023), which emphasize the positive contributions of emotional intelligence to individuals’ psychological well-being. Furthermore, this study further supports the notion that individuals with high emotional intelligence are more effective in analytical thinking and problem-solving skills, as stated by Cassady et al. (2022). Similar to previous studies highlighting the positive psychological outcomes of martial arts training, such as increased self-control, resilience, and emotional expression (Köroǧlu et al., 2025; Şahin et al., 2025), the current findings suggest that enhancing emotional intelligence may likewise foster mental well-being and adaptive coping skills.

However, mental well-being was found not to play a mediating role in the relationship between emotional intelligence and problem solving. This finding is consistent with Lu et al.’s (2024) study, which suggests that while mental well-being enhances an individual’s overall psychological functioning, it may not directly trigger problem-solving behaviors. Therefore, it can be argued that mental well-being alone does not have a decisive effect on problem-solving skills, rather, the direct effect of emotional intelligence is evident.

In light of these results, it is important to adopt an approach that focuses on the development of emotional intelligence in the design of psychoeducational programs for university students. Especially in the face of increased psychological stress in the post-pandemic period, supporting students’ emotional awareness, self-regulation, and empathy skills can strengthen both their well-being and their coping strategies in their academic and social lives (Zhou and Yao, 2020).

However, this study also has some limitations. The research was limited to students in the faculty of sports sciences, and examining student groups from different academic disciplines is important for general validity. Furthermore, because the study used a cross-sectional design, cause-and-effect relationships should be interpreted with caution.

Further research could examine in detail the interactions between different sub-dimensions of emotional intelligence (e.g., self-regulation, empathy, motivation) and problem-solving strategies. Furthermore, longitudinal studies could assess the effects of emotional intelligence over time and test the effectiveness of psychological interventions. This could contribute to both theoretical knowledge and practical psychological counseling processes.

### 4.1 Limitations

This study has several limitations. First, the fact that the sample consisted solely of university students studying at a faculty of sports sciences limits the generalizability of the results to other age groups, different educational fields, or different cultural contexts. The limited data collection method used in the study, limited to self-reported surveys and measurements, may pose the risk of social desirability or misrepresentation in participants’ responses. The use of a cross-sectional design in the study prevents the determination of causal relationships between variables; only correlational findings can be obtained. The predominantly male gender distribution among participants may lead to an incomplete assessment of gender-related differences in the analyses. Considering these limitations, the findings should be interpreted with caution, and future studies should attempt to address these limitations by using larger and more diverse samples, longitudinal designs, and control groups.

### 4.2 Recommendations

- Increasing sample diversity: Future studies can include participants from different universities, different age groups, and different fields of study, increasing the generalizability of the findings.
- Using a longitudinal design: By conducting longitudinal studies instead of a cross-sectional design, causal relationships between variables can be examined more reliably and changes over time can be observed.
- Controlling for additional external factors: By controlling for participants’ lifestyle and environmental factors, such as sleep patterns, eating habits, stress levels, or physical activity intensity, the accuracy and internal validity of the results can be increased.
- Diversifying data collection methods: Using objective measurements, biometric data, or observational methods in addition to self-reported surveys can strengthen data reliability and validity.

## Data Availability

The datasets presented in this study can be found in online repositories. The names of the repository/repositories and accession number(s) can be found in the article/supplementary material.
